# A combination of the QuantiFERON-TB Gold In-Tube assay and the detection of adenosine deaminase improves the diagnosis of tuberculous pleural effusion

**DOI:** 10.1038/emi.2016.80

**Published:** 2016-08-03

**Authors:** Yuanyuan Liu, Qinfang Ou, Jian Zheng, Lei Shen, Bingyan Zhang, Xinhua Weng, Lingyun Shao, Yan Gao, Wenhong Zhang

**Affiliations:** 1 Department of Infectious Diseases, Huashan Hospital, Fudan University, Shanghai 200040, China; 2 Department of Pulmonary Diseases, Wuxi No. 5 People's Hospital, Wuxi 214005, Jiangsu Province, China; 3 Key Laboratory of Medical Molecular Virology, Ministry of Education and Health, Shanghai Medical College, Fudan University, Shanghai 200032, China

**Keywords:** differential diagnosis, malignant pleural effusion, pleural ADA, QFT-GIT, tuberculous pleural effusion

## Abstract

The differential diagnosis of tuberculous pleural effusion (TPE) and malignant pleural effusion (MPE) remains difficult despite the availability of numerous diagnostic tools. The current study aimed to evaluate the performance of the whole blood QuantiFERON-TB Gold In-Tube (QFT-GIT) assay and conventional laboratory biomarkers in differential diagnosis of TPE and MPE in high tuberculosis prevalence areas. A total of 117 patients with pleural effusions were recruited, including 91 with TPE and 26 with MPE. All of the patients were tested with QFT-GIT, and the conventional biomarkers in both blood and pleural effusion were detected. The level of antigen-stimulated QFT-GIT in the whole blood of TPE patients was significantly higher than that of MPE (2.89 vs 0.33 IU/mL, *P*<0.0001). The sensitivity and specificity of QFT-GIT for the diagnosis of TPE were 93.0% and 60.0%, respectively. Among the biomarkers in blood and pleural effusion, pleural adenosine deaminase (ADA) was the most prominent biomarker, with a cutoff value of 15.35 IU/L. The sensitivity and specificity for the diagnosis of TPE were 93.4% and 96.2%, respectively. The diagnostic classification tree from the combination of these two biomarkers was 97.8% sensitive and 92.3% specific. Ultimately, the combination of whole blood QFT-GIT with pleural ADA improved both the specificity and positive predictive value to 100%. Thus, QFT-GIT is not superior to pleural ADA in the differential diagnosis of TPE and MPE. Combined whole blood QFT-GIT and pleural ADA detection can improve the diagnosis of TPE.

## INTRODUCTION

Tuberculous pleural effusion (TPE) is the most frequent manifestation of extra-pulmonary tuberculosis, accounting for ~5% of all forms of tuberculosis, and is the leading cause of pleural effusion in some high tuberculosis prevalence areas.^1, 2, 3, 4^ Malignant pleural effusion (MPE) is another major cause of lymphocyte-predominant exudative pleural effusion in developing countries. Although they present similar clinical manifestations, the prognosis and therapy of TPE and MPE are quite different. Thus, rapid and precise diagnostic tools for the differential diagnosis of pleural effusions are very important.

However, the differential diagnosis for TPE and MPE still remains a challenge. The gold standard for the diagnosis of TPE is still the detection of *Mycobacterium tuberculosis* (*M. tuberculosis*) in the pleural effusion, pleura tissue and/or respiratory specimens. Unfortunately, the positive rate is relatively low due to the paucity of mycobacteria in the pleural fluid. Less than 5% of the pleural fluid is smear positive, and the sensitivity of pleural fluid culture is only 24%–58%, which requires weeks to achieve.^5, 6, 7^ The performance of pleural biopsy has historically been considered the most reliable method to confirm the diagnosis. However, pleural biopsy is an invasive operation, and pleural tissue sampling is more difficult than simple thoracocentesis. A variety of pleural biomarkers have been proposed to assist in the diagnosis of TPE, and recent meta-analyses reveal that adenosine deaminase (ADA) and IFN-γ appear to be relatively accurate for TPE.^8^ Regarding MPE, a cytological examination of the pleural effusion is the main diagnostic method, but the diagnostic sensitivity varies between 30% and 60%, which can not meet the clinical needs.^9^

Recently, interferon-gamma release assays (IGRAs) have shown their superior diagnostic performance in the diagnosis of tuberculosis.^10, 11, 12^ The QFT-GIT test uses an enzyme-linked immunosorbent assay to measure the amount of IFN-γ released in response to specific *M. tuberculosis* antigens. The specific *M. tuberculosis* antigens are early secretory antigenic target-6 (ESAT-6), culture filtrate protein 10 (CFP-10) and TB 7.7, which are present in all *M. tuberculosis* and are able to stimulate the measurable release of IFN-γ in most infected persons, but are absent from bacillus Calmette-Guérin (BCG) vaccine strains and most nontuberculous mycobacteria.^13^ In 2008, QFT-GIT became the second IGRA approved by the US Food and Drug Administration (FDA) as a tool for diagnosing *M. tuberculosis* infection. The diagnostic utility of QFT-GIT in TPE has been evaluated in several studies, showing highly variable sensitivities and specificities.^14, 15^ Our study showed that the sensitivity and specificity of QFT-GIT were 93.1% (54/58) and 90.0% (18/20), respectively.^16^ However, the data on the assessment of QFT-GIT in the differential diagnosis of TPE and MPE are limited.

In this study, we evaluated the diagnostic value of whole blood QFT-GIT for the differential diagnosis of TPE and MPE in the TB-pandemic and BCG-covered regions. We also compared the blood and pleural effusion biomarkers with QFT-GIT to determine whether the differential diagnostic performance of QFT-GIT could be improved by combining it with the biomarkers.

## MATERIALS AND METHODS

### Study population

This study was conducted from January 2011 to September 2013 in Wuxi No. 5 People's Hospital. The Institutional Review Board (IRB) of Huashan Hospital Fudan University approved the study, and informed written consent was obtained from all the participants. A total of 138 patients with pleural effusion were enrolled, and all of them were aged 12 and older. All patients had a history taken and physical examination performed, along with routine investigations, including testing for HIV infection, chest radiography, and blood and pleural effusion examinations.

After providing informed consent, all of the patients were assessed with whole blood QFT-GIT upon enrollment and underwent thoracentesis, with 50 mL of pleural fluid collected. The laboratory data included cell counts, differential counts, the lactate dehydrogenase (LDH), ADA, carcinoembryonic antigen (CEA) and cancer antigen 125 (CA125) levels, and cytologic observation of malignant cells in the blood and pleural fluid. Acid-fast staining of smears and mycobacterial cultures of sputum and pleural fluid were performed. For accurate characterization of the disease, pleural biopsies and/or bronchoscopy examinations were also performed.

To minimize the effect of the anti-TB treatment, only patients who received standard anti-TB therapy for <1 week were included in the study. Patients with HIV infection and those who were severely immunocompromised or were receiving immunosuppressive drugs were excluded.

### Diagnostic criteria for the pleural effusions

The pleural effusions were first diagnosed as exudates using Light's criteria. According to Moon *et al*,^17^ confirmed tuberculous pleurisy was diagnosed by the presence of *M. tuberculosis*-positive cultures in the pleural effusion and/or confirmed TB infection by pleural biopsy. Probable tuberculous pleurisy was diagnosed using one of the following criteria: *M. tuberculosis*-positive culture in sputum, *M. tuberculosis*-positive culture in other biologic specimens or positive response to antituberculosis medication without other possible causes of pleural effusion. Malignant pleural effusion was diagnosed when there was positive pleural fluid cytology and/or positive pleural biopsy histology.

### QuantiFERON TB Gold In-Tube Test

The QFT-GIT test (Cellestis Ltd, Carnegie, Australia) was performed according to the manufacturer's instructions. Briefly, 1 mL of whole blood was drawn into three QFT tubes coated with saline (Nil control), a peptide cocktail containing the ESAT-6, CFP-10 and TB7.7 proteins (TB Antigen), or PHA (mitogen control), respectively, and incubated at 37 °C as soon as the blood was collected (within 8 h after collection). Following a 20-h incubation period, the tubes were centrifuged, and the plasma was harvested from each tube to determine the concentration of IFN-γ. QFT-GIT tests were regarded as positive if the antigen-stimulated response of IFN-γ (TB Ag-Nil) was ⩾0.35 IU/mL (17.5 pg/mL), negative if the mitogen-stimulated response (Mitogen-Nil) was ⩾0.5 IU/mL (25 pg/mL) and the antigen-stimulated response was <0.35 IU/mL or indeterminate if both the mitogen-stimulated and antigen-stimulated responses were <0.35 IU/mL or the unstimulated response (Nil) was >8 IU/mL (400 pg/mL). The IFN-γ concentration was presented as pg/mL to facilitate comparisons with the other biomarkers detected. One International unit of IFN-γ corresponds to 50 pg/mL (NIBSC, Potters Bar, UK).

### Statistical analysis

The data from both groups were compared using the non-parametric Mann–Whitney test and Chi-square test or Fisher's exact test. The diagnostic accuracies of the tests were evaluated using receiving operating characteristic (ROC) curves. The cutoff values were estimated at various sensitivities and specificities and determined at the maximum Youden's index (YI). The diagnostic classification tree was developed by considering all the biomarkers using the R program, with a 15-fold cross-validation. A two-tailed *P*<0.05 was considered statistically significant. The statistical analysis was performed using GraphPad Prism V5.03 software (GraphPad, San Diego, CA, USA).

## RESULTS

### Clinical characteristics of study participants

**Figure 1:**
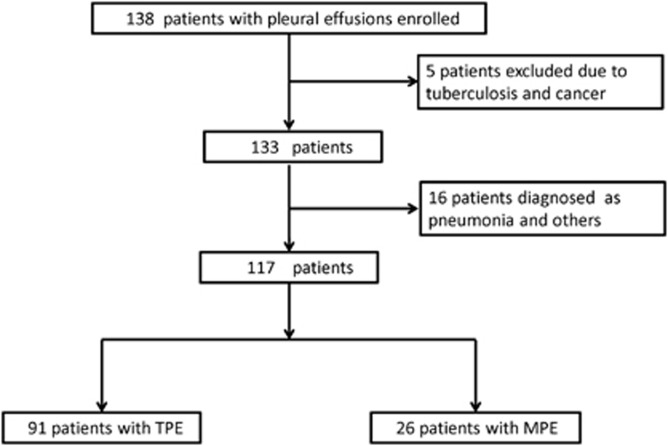
Recruitment and diagnostic classification of all participants.

Of the 138 patients enrolled in this study, five were excluded due to a diagnosis of tuberculosis with cancer and 16 patients were excluded due to a diagnosis of pneumonia or other diseases. Based on the final diagnosis, 117 patients with pleural effusion were divided into two groups: the TPE group (*n*=91) and the MPE group (*n*=26) (Figure 1). None of the patients was infected with HIV. The demographic and clinical characteristics of patients in this study were shown in Table 1. The median age of the enrolled patients was 48 years. The MPE cases were older than the patients with TPE (65 vs 45 years, *P*<0.0001). There were 86 males out of the 117 patients (73.5%), and 90 individuals (76.9%) had received a BCG vaccination. Among the 91 TPE patients, 37 patients were diagnosed with confirmed TB pleurisy with culture/biopsy evidence: eight by positive pleural culture, one by positive pleural culture and AFB smear, four by positive pleural culture and pleural biopsy, and 24 by pleural biopsy. The remaining 54 patients were diagnosed with probable TB pleurisy with clinical evidence: 25 by sputum culture positive for *M. tuberculosis* and 29 by a positive response to anti-TB treatment. All of the 26 patients with MPE were histologically diagnosed by thoracoscopy or bronchoscopy.

### Diagnostic performance of QFT-GIT

**Figure 2:**
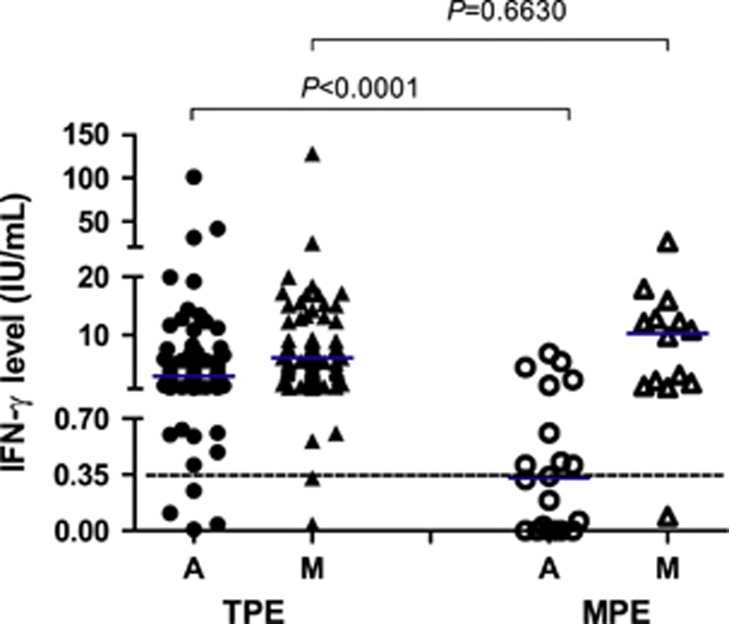
TB antigen-stimulated (**a**) and mitogen-stimulated (M) IFN-γ levels in the TPE (*n*=86) and MPE (*n*=20) groups. The short transverse line represents the median level. The dotted transverse line represents the cutoff value for QFT-GIT.

The median level of TB antigen-stimulated IFN-γ in the QFT-GIT test was significantly higher in the TPE group than in the MPE group (2.89 IU/mL vs 0.33 IU/mL; *P*<0.0001), whereas the levels of PHA-stimulated IFN-γ were comparable in both groups (*P*=0.6630) (Figure 2). The positive QFT-GIT rates in the TPE group and the MPE group were 93.0% (80/86) and 40.0% (8/20), respectively, with statistically significant differences (*P*<0.0001).

**Figure 3:**
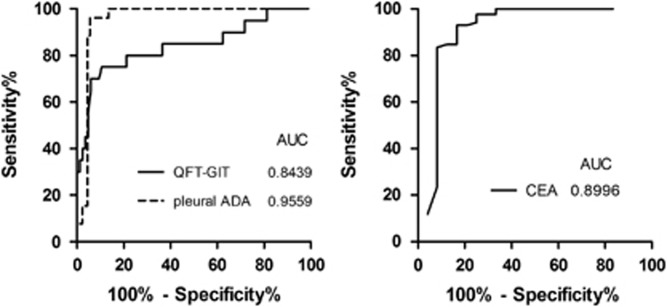
The ROC curves of QFT-GIT, pleural ADA and pleural CEA for the differential diagnosis of TPE and MPE.

The ROC curve analysis showed that the area under the curve (AUC) of the whole blood QFT-GIT for discriminating TPE from MPE was 0.8439 (Figure 3). The sensitivity and specificity of QFT-GIT for diagnosing TPE were 93.0% (95% CI: 84.9%–97.1%) and 60.0% (95% CI: 36.4%–80.0%), respectively. The positive predictive value (PPV), negative predictive value (NPV) and accuracy of QFT-GIT were 90.9%, 66.7% and 86.8%, respectively (Table 2). There was no significant difference in the sensitivity between confirmed TPE cases (91.4%, 32/35) and probable TPE cases (94.1%, 48/51; *P*>0.05).

### Comparison of biomarkers in blood and pleural effusion

To identify predictive features for the diagnosis of TPE or MPE, we compared the admission variables of the patients in both groups, including the results of the blood tests and pleural effusion tests. As shown in Table 3, the median lymphocyte percentages in the pleural fluid of the TPE group and MPE group were 83.3% and 81.0%, respectively, which were present in the lymphocyte-predominant exudates. Blood lymphocytes %, pleural lymphocytes %, blood CA125 levels, pleural CA125 levels and pleural gravity were not different between the two groups by univariate analysis. Ten variables were identified that exhibited a statistically significant difference between the two groups (Table 3).

The diagnostic potential of the ten biomarkers in the differential diagnosis of TPE and MPE was assessed with the ROC curve, and only the AUC of pleural ADA and pleural CEA were higher than QFT-GIT (Figure 3).

### Diagnostic comparisons of QFT-GIT, pleural ADA and pleural CEA

The ROC curves of the three diagnostic tests for distinguishing TPE from MPE were shown in Figure 3. The AUCs of the pleural ADA, pleural CEA and QFT-GIT were 0.9568 (95% CI: 0.9198–0.9947), 0.8996 (95% CI: 0.7960–1.003) and 0.8439 (95% CI: 0.7273–0.9568), respectively. According to the ROC curve, the optimal cutoff values for the pleural ADA and pleural CEA were determined to be 15.35 IU/L and 3.450 ng/mL, respectively. The corresponding sensitivity, specificity, PPV, NPV and accuracy were summarized in Table 2. With a cutoff value of 15.35 IU/L, the sensitivity and specificity of pleural ADA for diagnosis of TPE were 93.4% (95% CI: 85.7%–97.3%) and 96.2% (95% CI: 78.4%–99.8%), respectively, and the diagnostic accuracy was 94.0%. The PPV of pleural ADA was up to 98.8%. In addition, as shown in Table 2, with a cutoff value of 3.450 ng/mL, the sensitivity and specificity of pleural CEA for diagnosis of MPE were 92.9% (95% CI: 84.7%–97.1%) and 83.3% (95% CI: 61.8%–94.5%), respectively, and the accuracy was 90.8%. Furthermore, our data indicated that pleural ADA yielded better specificity, PPV, NPV and accuracy than QFT-GIT, but equivalent sensitivity (93.4% vs 93.0%, *P*=0.0990) for the diagnosis of TPE. Similarly, the specificity, PPV, NPV and accuracy of pleural CEA for the diagnosis of MPE were also superior to QFT-GIT, but the sensitivity was equivalent (92.9% vs 93.0%, *P*=0.3319).

### Improved performance by combining QFT-GIT and ADA

**Figure 4:**
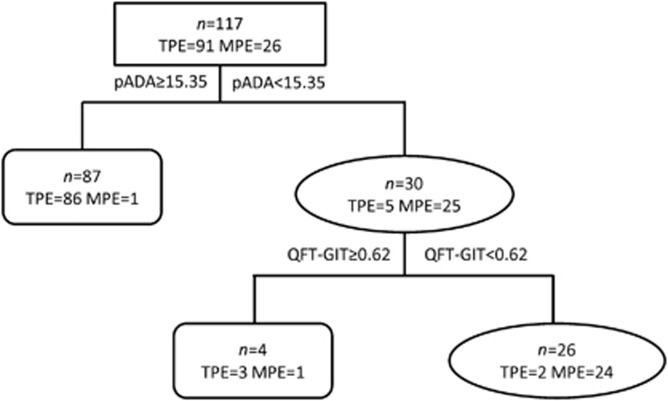
The combination of QFT-GIT and pleural ADA provides a better discrimination between TPE and MPE. The sensitivity and specificity of the diagnostic classification tree were 97.8% and 92.3%, respectively, and the accuracy was 96.6%.

We further assessed whether the diagnostic efficiency could be improved when the QFT-GIT test was combined with other biomarkers. We subjected the above 10 biomarkers to decision-tree analysis to identify the ideal biomarker combination and to optimize the discrimination between TPE and MPE. The analysis indicated that the combination of pleural ADA with QFT-GIT provided the best predictive capacity (Figure 4). Using the ROC assay, the cutoff value of QFT-GIT was 0.62 IU/mL. The sensitivity of the diagnostic classification tree was 97.8%, as 89 out of 91 TPE cases were correctly identified, and the specificity was 92.3%, as only 2 out of 26 MPE cases were incorrectly identified as TPE.

We subsequently evaluated the diagnostic utility of the combination of QFT-GIT, pleural ADA and pleural CEA. Parallel tests and serial tests were introduced into our analysis. In the parallel test of combined ‘QFT or ADA', the sensitivity and NPV increased to 98.9% (95% CI: 93.2%–99.9%) and 94.4% (95% CI: 70.6%–99.7%), respectively, compared with the sensitivity and NPV of QFT-GIT of 93.0% and 66.7%. Meanwhile, the accuracy of ‘QFT or ADA' increased from 86.8% to 91.5%. On the other hand, the serial test of combined ‘QFT and ADA' yielded the highest specificity of 100% (95% CI: 84.0%–100%) and PPV of 100% (95% CI: 94.1%–100% Table 4). However, the combination of QFT and/or CEA did not have better diagnostic utility than the combination of QFT and/or ADA.

## DISCUSSION

Tuberculous and malignant pleural effusions are the leading causes of pleural effusion in China, which is a TB endemic and BCG-vaccinated region. Although both are lymphocyte-predominant exudative pleural effusions, the clinical treatments and prognosis vary significantly. A positive mycobacterial tuberculosis examination is the gold standard for the diagnosis of TPE. However, TPE is a delayed hypersensitivity to mycobacterial antigens in the pleural space, which often results in a negative microbiological analysis and lower rates of *M. tuberculosis-*positive cultures and smears from pleural effusions.^18, 19^ In our study, the rate of *M. tuberculosis*-positive cultures was 14.3% (13/91) and the rate of *M. tuberculosis*-positive smears was 1.1% (1/91) in tuberculous pleural effusions, which were far from the clinical requirements. In addition, a histopathological examination of the biopsy sample via thoracoscopy may improve the diagnostic sensitivity, specificity and accuracy of TPE.^20^ Thirty-two patients in our TPE group underwent thoracoscopy for biopsy of the pleura, with a positive rate of 87.5% (28/32). However, thoracoscopy is an invasive procedure that is not suitable or available for all patients.

The IGRAs have been used to diagnose active TB and latent TB infections, and the diagnostic value of IGRAs in TPE has been examined in several studies. Chung *et al.* evaluated 97 subjects, 54 of whom had been classified as having TPE and 43 as having non-TPE. With the use of the QFT-GIT assay in these patients, the sensitivity was 76.9%, which was significantly higher than the sensitivity of the tuberculin skin test (TST; 72.5% *P*=0.003).^15^ Our previous data showed that the sensitivity and specificity of whole blood QFT-GIT in diagnosing TPE were 93.1% and 90.0%, whereas those of TST were 68.5% and 86.7%, respectively.^16^ Thus, QFT-GIT was superior to TST for diagnosing TPE. However, other studies showed that the QFT-GIT assay of the peripheral blood or adapted pleural fluid was not very accurate for the diagnosis of TPE.^21^

However, the usefulness of QFT-GIT in differential diagnosis of TPE and MPE remains unclear. The QFT-GIT assay is used to measure the level of TB-specific IFN-γ in whole blood. As expected, in this study, the positive rate of the whole blood QFT-GIT was significantly higher in TPE group than in the MPE group (93.0% vs 40.0%, *P*<0.0001). Moreover, the sensitivity, specificity and accuracy of QFT-GIT for the differential diagnosis of TPE were 93.0%, 60.0% and 86.8%, respectively. Therefore, QFT-GIT was more sensitive and rapid than conventional microbiological tests, but exhibited poor specificity in our setting because of the high prevalence of latent TB infection.

Previous studies showed that a series of biomarkers have important roles in the differential diagnosis of tuberculosis and malignant pleural effusions.^22, 23^ ADA has been reported to be a sensitive and specific marker for diagnosing TPE. ADA is an essential enzyme in the metabolism of purine nucleosides, and its activity correlates with T-lymphocyte infiltration in the pleura and pleural fluid. Tuberculosis is cell immune response mediated by T lymphocytes; therefore, the ADA level also increases accordingly. Our results showed that the pleural ADA levels of the TPE group were significantly higher than those of the MPE group. Moreover, the AUC of the pleural ADA, pleural CEA and whole blood QFT-GIT accounted for the top three blood and pleural biomarkers (Table 3), of which ADA had the largest AUC (Figure 3). Using a 15.35 IU/L cutoff point, the sensitivity, specificity, PPV, NPV and accuracy of ADA were higher than those of QFT-GIT and CEA. However, according to the ROC curves, the optimal cutoff level of ADA is lower than the reported cutoff value (30-60 IU/L). One possible explanation for the difference is that the TPE patients in the present study are older than those in previous studies. According to the literature, the ADA level is negatively correlated with age.^24^ However, we did not find that the pleural ADA level was negatively correlated with age. In addition, some research suggests that the ADA activity was reduced when a rat model was exposed to smoke,^25^ but we did not record the smoking history of the participants in our study.

CEA has been studied extensively and has been found to have value in diagnosing MPE.^26^ In our study, pleural CEA had a sensitivity of 92.9% and a specificity of 83.3% for the diagnosis of MPE with the cutoff value of 3.450 ng/mL. Moreover, the diagnostic accuracy of pleural CEA was higher than QFT-GIT.

A diagnostic classification tree was developed in our study, and the combination of pleural ADA and QFT-GIT provided the best predictive capacity, with a sensitivity of 97.8% and a specificity of 92.3%. We further assessed the diagnostic performance when the pleural ADA was combined with the QFT-GIT. The combination of ADA with QFT-GIT increased the specificity to 100%, and the PPV simultaneously increased to 100%. Meanwhile, the specificity and PPV increased to 95.7% and 98.6% for the combination of QFT-GIT and CEA, compared with QFT-GIT at 60.0% and 90.9%, respectively. However, the diagnostic accuracy of the combination of QFT-GIT and CEA was not superior to ADA.

This study had some limitations. The primary limitation is that we did not include a pleural fluid QFT-GIT assay for the diagnosis of TPE. In fact, a previous study indicated that the diagnostic performance of the pleural fluid QFT-GIT did not appear to be better than whole blood QFT-GIT for TPE diagnosis.^27^ The second limitation is the small number of MPE patients in the study, and a large-scale study is needed in the future.

In summary, QFT-GIT is not superior to pleural ADA or pleural CEA in the differential diagnosis of TPE and MPE. The diagnostic classification tree with QFT-GIT and pleural ADA was 97.8% sensitive and 92.3% specific. Combinations of QFT-GIT and ADA yielded a specificity of 100% and PPV of 100%. However, in clinical practices, the discriminating diagnosis must be determined by taking many factors into account rather than the use of any single method to avoid any misdiagnose of diseases.

## Figures and Tables

**Table 1:** The demographic and clinical characteristics of study participants

	**Total**	**TPE**	**MPE**
Patients, *n*	117	91	26
Median age (range)	48 (12–89)	45 (12–84)	65 (39–89)
Male (%)	86 (73.5%)	67 (73.6%)	19 (73.1%)
BCG vaccinated, *n* (%)	90 (76.9%)	75 (82.4%)	15 (57.7%)
Positive, sputum, AFB smear or culture, *n* (%)	—	25/89 (28.1%)	0

*M. tuberculosis detection in pleural effusion*			
Positive AFB smear, *n* (%)	—	1/89 (1.1%)	0
Positive culture, *n* (%)	—	13/83 (15.7%)	0
Confirmed TB by pleural biopsy, *n* (%)	—	28/32 (87.5%)	0

**Table 2:** Diagnostic comparison of QFT-GIT, ADA and CEA for the differential diagnosis of TPE and MPE

**Assays (cutoff value)**	**Sensitivity (%) (95% CI)**	**Specificity (%) (95% CI)**	**PPV (%) (95% CI)**	**NPV (%) (95% CI)**	**Accuracy (%)**
Pleural ADA (15.35 IU/L)	93.4 (85.7–97.3)	96.2 (78.4–99.8)	98.8 (92.8–99.9)	80.6 (61.9–91.9)	94.0
Pleural CEA (3.450 ng/mL)	92.9 (84.7–97.1)	83.3 (61.8–94.5)	95.2 (87.5–98.4)	76.9 (55.9–90.2)	90.8
QFT-GIT	93.0 (84.9–97.1)	60.0 (36.4–80.0)	90.9 (82.4–95.7)	66.7 (41.2–85.6)	86.8

Abbreviations: adenosine deaminase, ADA; carcinoembryonic antigen, CEA; negative predictive value, NPV; positive predictive value, PPV; QuantiFERON-TB Gold In-Tube, QFT-GIT.

**Table 3:** Comparisons of biomarkers in blood and pleural effusion between patients with TPE and MPE

	**TPE median (90% range)**	***n***	**MPE median (90% range)**	***n***	***P***	**AUC**
*Blood tests*						
WBC (10^9^/L)	6.60 (4.46–9.83)	90	8.95 (5.17–16.58)	26	0.0008	0.7171
Neutrophils (%)	66.05 (56.92–77.56)	90	72.30 (60.60–88.04)	26	0.0078	0.6718
Lymphocytes (%)	23.60 (13.64–34.40)	90	20.20 (4.77–32.31)	26	0.0761	0.6145
ADA (IU/L)	13.70 (9.08–20.6)	57	7.20 (4.61–17.05)	12	0.0005	0.8224
CA125 (U/L)	104.8 (31.8–295.5)	85	101.6 (24.8–564.2)	25	0.4348	0.5515
CEA (ng/mL)	1.4 (0.6–2.9)	86	4.3 (0.7–722.3)	26	<0.0001	0.7983
QFT-GIT (IU/mL)	2.89 (0.61–12.94)	86	0.33 (0.00–5.28)	20	<0.0001	0.8439

*Effusion tests*						
LDH (IU/L)	478.6 (288.9–924.2)	88	280.0 (152.0–949.0)	26	0.0013	0.7087
ADA (IU/L)	35.30 (18.60–56.50)	89	6.40 (2.56–14.85)	26	<0.0001	0.9559
Protein (g/L)	47.25 (39.46–53.58)	90	43.60 (30.90–51.57)	26	0.0024	0.6959
Specific gravity	1.020 (1.016–1.021)	89	1.020 (1.015–1.022)	26	0.6398	0.5303
WCC (10^6^/L)	3000 (990–6010)	88	1150 (400–2821)	26	<0.0001	0.8201
Lymphocytes (%)	83.30 (48.90–91.28)	88	81.00 (61.40–86.60)	26	0.2652	0.5721
CA125 (U/L)	780.2 (44.0–1000.0)	79	948.0 (136.4–1000.0)	21	0.2604	0.5802
CEA (ng/mL)	1.1 (0.5–2.9)	81	126.9 (1.3–1500.0)	23	<0.0001	0.8996

Abbreviations: adenosine deaminase, ADA; cancer antigen 125, CA125; carcinoembryonic antigen, CEA; lactate dehydrogenase, LDH; malignant pleural effusion, MPE; QuantiFERON-TB Gold In-Tube, QFT-GIT; tuberculous pleural effusion, TPE; white cell count, WCC.

**Table 4:** Diagnostic utility of QFT-GIT, ADA, CEA and their combinations for the differential diagnosis of TPE and MPE

**Assays (cutoff value)**	**Sensitivity (%) (95% CI)**	**Specificity (%) (95% CI)**	**PPV (%) (95% CI)**	**NPV (%) (95% CI)**	**Accuracy (%)**
QFT-GIT or ADA	98.9 (93.2–99.9)	65.4 (44.4–82.1)	90.9 (83.0–95.5)	94.4 (70.6–99.7)	91.5
QFT-GIT and ADA	87.5 (78.3–93.3)	100.0 (84.0–100)	100.0 (94.1–100)	70.3 (52.8–83.6)	90.4
QFT-GIT or CEA	97.8 (91.5–99.6)	57.9 (34.0–78.9)	91.8 (83.9–96.1)	84.6 (53.7–97.3)	90.9
QFT-GIT and CEA	86.7 (77.1–92.9)	95.7 (76.0–99.8)	98.6 (91.6–99.9)	66.7 (48.1–81.4)	88.7

Abbreviations: adenosine deaminase, ADA; carcinoembryonic antigen, CEA; QuantiFERON-TB Gold In-Tube, QFT-GIT.
